# *miR-138-5p* contributes to cell proliferation and invasion by targeting Survivin in bladder cancer cells

**DOI:** 10.1186/s12943-016-0569-4

**Published:** 2016-12-15

**Authors:** Rong Yang, Minghui Liu, Hongwei Liang, Suhan Guo, Xu Guo, Min Yuan, Huibo Lian, Xiang Yan, Shiwei Zhang, Xi Chen, Feng Fang, Hongqian Guo, Chenyu Zhang

**Affiliations:** 1 Department of Urology, The Affiliated Drum Tower Hospital of Nanjing University Medical School, 321 Zhongshan Road, Nanjing, Jiangsu 210008 China; 2 Jiangsu Engineering Research Center for microRNA Biology and Biotechnology, State Key Laboratory of Pharmaceutical Biotechnology, School of Life Sciences, Nanjing University, Nanjing, 210093 China; 3 Department of Pharmacology, Nanjing Medical University, 101 longmian Avenue, Nanjing, Jiangsu 211166 China; 4 School of Public Health, Nanjing Medical University, 101 longmian Avenue, Nanjing, Jiangsu 211166 China

**Keywords:** Bladder cancer, miR-138-5p, Survivin, post-transcriptional regulation

## Abstract

**Background:**

Survivin (encoded by the gene BIRC5) plays an important role in the carcinogenesis of bladder cancer. Identifying miRNAs that target Survivin in the setting of bladder cancer will help to develop Survivin-based therapies for bladder cancer.

**Methods:**

The expression levels of miR-138-5p and Survivin protein were measured in 12 resected bladder cancer specimens. The correlation between miR-138-5p and Survivin was further examined by evaluating Survivin expression in human bladder cancer cell lines that either overexpressed or knocked down miR-138-5p. A luciferase reporter assay was performed to test the direct binding of miR-138-5p to the target gene BIRC5. We also investigated the biological role of miR-138-5p targeting to Survivin in bladder cancer cell lines both *in vivo* and *in vitro*.

**Results:**

In this study, we found that the Survivin protein was either absent or weakly expressed in normal adjacent tissues and consistently up-regulated in bladder cancer tissues; however, the mRNA levels did not vary as much, suggesting that a post-transcriptional mechanism was involved. Because microRNAs are powerful post-transcriptional regulators of gene expression, we used bioinformatic analyses to search for microRNAs that could potentially target BIRC5 in the setting of bladder cancer. We identified 2 specific targeting sites for miR-138-5p in the 3′ untranslated region (3′-UTR) of BIRC5. We further identified an inverse correlation between miR-138-5p and Survivin protein levels in bladder cancer tissue samples. By overexpressing or knocking down miR-138-5p in bladder cancer cells, we experimentally confirmed that miR-138-5p directly recognizes the 3′-UTR of the BIRC5 transcript and regulates Survivin expression. Furthermore, the biological consequences of the targeting of BIRC5 by miR-138-5p were examined *in vitro* via cell proliferation and invasion assays and *in vivo* using a mouse xenograft tumor model. We demonstrated that BIRC5 repression by miR-138-5p suppressed the proliferative and invasive characteristics of bladder cancer cells and that miR-138-5p exerted an anti-tumor effect by negatively regulating BIRC5 in a xenograft mouse model.

**Conclusions:**

Taken together, our findings provide the first clues regarding the role of miR-138-5p as a tumor suppressor in bladder cancer by inhibiting BIRC5 translation.

**Electronic supplementary material:**

The online version of this article (doi:10.1186/s12943-016-0569-4) contains supplementary material, which is available to authorized users.

## Background

Bladder cancer is the most common malignancy of the urogenital system and is one of the major causes of cancer-related death among Chinese patients. Based on statistics by Chen et al., an estimated 80,500 new cases of urinary bladder cancer would be diagnosed and approximately 32,900 deaths from bladder cancer were anticipated in China in 2015 [1]. Bladder cancer can be classified into two types according to the tumor invasion depth: non-muscle invasive tumor (70 ~ 80%) and muscle-invasive tumor (20 ~ 30%) [2]. Compared with non-muscle invasive tumors, muscle-invasive tumors are a highly aggressive disease. The 5-year survival rate for patients with non-muscle invasive bladder cancer is nearly 90%, whereas that for patients with muscle-invasive bladder cancer is approximately 60% [3]. Therefore, it is of great clinical significance to clarify the mechanisms underlying the aggressive progression of bladder cancer, which will help to identify specific molecular targets and develop more effective therapies for this disease.

Survivin (encoded by the gene BIRC5) is a key member of the inhibitor of apoptosis protein (IAP) family [4]. Dysregulation of Survivin is a typical signature of many cancers. Survivin is not present in normal bladder urothelium but was expressed in a high percentage of patients with bladder cancer [5]. A multicenter study found Survivin expression was associated with an elevated risk of bladder cancer recurrence and cancer-specific mortality [6]. Survivin and E-cadherin could help identify patients in the initial pTa stage of bladder cancer who were at risk of developing progressive disease [7]. The results above have led to the proposal of targeting Survivin as a promising alternative treatment for bladder cancer.

microRNAs (miRNAs) are endogenous small non-coding RNA molecules (19-22 nucleotides in length) that regulate protein coding gene expression by binding to the 3’ untranslated region (UTR) of mRNAs to yield an RNA-induced silencing complex [8]. Increasing evidence suggests that miRNAs are aberrantly expressed in various human cancers and that they play significant roles in cancer initiation, development, and metastasis [9]. Some highly expressed miRNAs could function as oncogenes by repressing tumor suppressors, whereas miRNAs expressed at lower levels could function as tumor suppressors by negatively regulating oncogenes [10]. miRNAs potently influence cellular activities through the regulation of extensive gene expression networks [11, 12]. Therapeutic modulation of a single miRNA may therefore simultaneously affect many pathways to achieve better clinical results. Survivin was reported to be post-transcriptionally regulated by several miRNAs in various tumors [13]. The identification of miRNAs that target Survivin in the setting of bladder cancer will help in the development of Survivin-based therapies for bladder cancer [14].

Although the dysregulation of miRNAs and Survivin plays important roles in the carcinogenesis of bladder cancer, there are limited reports about the correlation between Survivin and miRNAs in bladder cancer. In this study, we predicted that Survivin was a target of miR-138-5p. After measuring the expression levels of miR-138-5p and Survivin in human bladder cancer tissues and adjacent noncancerous tissue samples, we detected an inverse correlation between miR-138-5p expression and Survivin protein levels. Furthermore, we experimentally investigated the direct regulation of Survivin by miR-138-5p as well as the biological role of miR-138-5p targeting Survivin in human bladder cancer cell lines and in a mouse tumor xenograft model.

## Methods

### Cells and human tissues

The human bladder cancer cell lines T24 and J82 were purchased from the Shanghai Institute of Cell Biology at the Chinese Academy of Sciences (Shanghai, China). The cells were cultured in RPMI 1640 medium supplemented with 10% fetal bovine serum (FBS, Gibco, Carlsbad, CA, USA) in a humidified atmosphere containing 5% CO_2_. The bladder cancer specimens and paired normal adjacent tissues were obtained from patients undergoing a surgical procedure at the Affiliated Drum Tower Hospital of Nanjing University, School of Medicine (Nanjing, China). All the patients provided written consent, and the Ethics Committee from Nanjing University approved all aspects of this study. Tissue fragments were immediately frozen in liquid nitrogen at the time of surgery and stored at -80 °C.

### RNA isolation and quantitative RT-PCR

Total RNA was extracted from cultured cells and human tissues using TRIzol Reagent (Sigma, St. Louis, MO, USA) according to the manufacturer’s instructions. Assays to quantify miRNA levels were performed using TaqMan miRNA probes (Applied Biosystems, Foster City, CA, USA) according to the manufacturer’s instructions. Briefly, 1 μg of total RNA was reverse-transcribed into cDNA using AMV reverse transcriptase (TaKaRa, Dalian, China) and a stem-loop RT primer (Applied Biosystems). The reaction conditions were as follows: 16 °C for 30 min, 42 °C for 30 min, and 85 °C for 5 min. Real-time PCR was performed using a TaqMan PCR kit on an Applied Biosystems 7300 Sequence Detection System (Applied Biosystems). The reactions were incubated in a 96-well optical plate at 95 °C for 10 min followed by 40 cycles of 95 °C for 15 s and 60 °C for 1 min. All of the reactions were performed in triplicate. After the reaction, the cycle threshold (C_T_) data were determined using fixed threshold settings, and the mean C_T_ was determined from the triplicate PCRs. A comparative C_T_ method was used to compare each condition to the controls. The relative levels of miRNAs in the cells and tissues were normalized to the U6 levels. The amount of miRNA relative to the internal control U6 was calculated with the equation 2^-△△CT^, in which △△C_T_ = (C_T miR-138-5p_ - C_T__U6_) _tumor_ - (C_T__miR-138-5p_ - C_T__U6_) _control_. To quantify Survivin mRNA expression, 1 μg of total RNA was reverse-transcribed into cDNA using oligo dT and AMV reverse transcriptase (TaKaRa, Dalian, China) with the following cycling conditions: 16 °C for 30 min, 42 °C for 30 min, and 85 °C for 5 min. Next, real-time PCR was performed with the resulting RT product, SYBR Green Dye (Invitrogen) and specific primers for Survivin and GAPDH. The sequences of the primers were as follows: Survivin (sense), 5′- CAACCGGACGAATGCTTTT-3′; Survivin (antisense), 5′-AAGAACTGGCCCTTCTTGGA-3′; GAPDH (sense), 5′-CGAGCCACATCGCTCAGACA-3′; and GAPDH (antisense), 5’-GTGGTGAAGACGCCAGTGGA-3’. The reactions were incubated at 95 °C for 5 min followed by 40 cycles of 95 °C for 30 s, 60 °C for 30 s and 72 °C for 1 min. After the reactions were completed, the C_T_ values were determined by setting a fixed threshold. The relative amount of Survivin mRNA was normalized to GAPDH using the △△C_T_ method as described above.

### Protein extraction and Western blotting

The cells and tissues were lysed in ice-cold RIPA lysis buffer (Beyotime, Shanghai, China) supplemented with a protease and phosphatase inhibitor cocktail (Thermo Scientific 78440), incubated on ice for 30 min and then centrifuged for 10 min (12, 000 × g, 4 °C). The supernatant was collected, and the protein concentration was calculated using a Pierce BCA protein assay kit (Thermo Scientific, Rockford, IL, USA). The Survivin protein levels were analyzed by Western blotting with a monoclonal anti-human Survivin antibody (71G4B7, 2808 s. Cell Signaling Technology, MA, USA). The protein levels were normalized by probing the same blots with a GAPDH antibody (FL-335, sc-25778, Santa Cruz Biotechnology, CA, USA).

### Overexpression or knockdown of miR-138-5p

miR-138-5p overexpression was achieved by transfecting bladder cancer cell lines with an miRNA mimic (a synthetic RNA oligonucleotide duplex mimicking the miRNA precursor), and knockdown was achieved by transfecting a miRNA inhibitor (a chemically modified single-stranded antisense oligonucleotide designed to specifically target the mature miRNA). Synthetic mim-miR-138-5p, anti-miR-138-5p and the corresponding negative control scrambled RNAs (mim-scramble and anti-scramble) were purchased from GenePharma (Shanghai, China). T24 and J82 cells were seeded into 6-well plates using RPMI 1640 media supplemented with 10% FBS. The following day when the cells reached 60-70% confluency, the cells were transfected with Lipofectamine 2000 (Invitrogen) using Opti-MEM Reduced Serum Medium (Gibco, Carlsbad, CA, USA). Equal amounts of either mim-miR-138-5p or mim-scramble were used in each well. For miRNA knockdown, equal amounts of anti-miR-138-5p or anti-scramble were used. After 6 hours, the media was replaced with RPMI 1640 supplemented with 2% FBS. The cells were harvested 24 hours after transfection and subjected to analysis by quantitative RT-PCR or Western blotting.

### Plasmid construction and siRNA interference assay

A mammalian expression plasmid encoding the full-length human BIRC5 open reading frame (ORF) without the miR-138-5p-responsive 3′-UTR was purchased from Ribobio (Guangzhou, China). An empty plasmid served as the negative control. Three siRNA sequences targeting different sites of the human Survivin cDNA (si-Survivin) were designed and synthesized by Ribobio (Guangzhou, China). A scrambled siRNA that does not target the human Survivin cDNA was synthesized as a negative control. The siRNA sequences were as follows: si-Survivin #1, 5′-ACCGCATCTCTACATTCAA-3′ (sense); si-Survivin #2, 5′-TCCGGTTGCGCTTTCCTTT-3′ (sense); and si-Survivin #3, 5′-ACCACCGCATCTCTACATT-3′ (sense). Either the Survivin overexpression plasmid or the Survivin siRNAs were transfected into T24 cells using Lipofectamine 2000 (Invitrogen) according to the manufacturer’s instructions. Total RNA and protein were isolated 24 hours after transfection. The Survivin mRNA and protein expression levels were assessed by quantitative RT-PCR and Western blotting, respectively. The siRNA sequence with the best interfering effect (si-Survivin#1) was selected and used for all subsequent experiments.

### Luciferase reporter assay

To test the direct binding of miR-138-5p to the target gene BIRC5, a luciferase reporter assay was performed as previously described. [15] A sequence containing the presumed miR-138-5p binding site was designed from the human BIRC5 3′-UTR. The sequence was inserted into the p-MIR-reporter plasmid (Ambion). The insertion was confirmed to be correct by sequencing. To test the binding specificity, the sequences that interacted with the miR-138-5p seed sequence were mutated (site 1 from CACCAGC to GTGGTCG, and site 2 from ACCAGCA to TGGTCGT), and the mutant BIRC5 3′-UTR was inserted into an equivalent luciferase reporter. For the luciferase reporter assays, HEK293 cells were cultured in 24-well plates, and each well was transfected with 0.4 μg of firefly luciferase reporter plasmid, 0.4 μg of a β-galactosidase (β-gal) expression plasmid (Ambion), and equal amounts (20 pmol) of mim-miR-138-5p, anti-miR-138-5p, or the scrambled negative control RNAs using Lipofectamine2000 (Invitrogen). The β-gal plasmid was used as a transfection control. Twenty-four hours after transfection, the cells were assayed using a luciferase assay kit (Promega, Madison, WI, USA).

### Cell proliferation assay

The proliferation of T24 cells was determined using the Cell Counting Kit-8 (CCK-8, Dojindo) according to the manufacturer’s instructions. T24 cells were plated at a density of 5 × 10^3^ cells per well in 96-well plates and incubated overnight in RPMI 1640 medium supplemented with 10% FBS. The next day, the T24 cells were transfected with mim-miR-138-5p, anti-miR-138-5p, BIRC5 siRNA and/or the overexpression plasmid, and the medium was replaced with RPMI 1640 medium supplemented with 2% FBS. At 12, 24, 36 and 48 hours after transfection, 10 μL of CCK-8 reagent was added to the test well and incubated for 3 hours. The absorbance was measured at a wavelength of 450 nm.

### Cell invasion assay

The invasive ability of T24 cells transfected with either mimics or inhibitors (mim-miR-138-5p or anti-miR-138-5p, respectively), BIRC5 siRNA and/or the overexpression plasmid was tested in a Transwell Boyden chamber (6.5 mm; Costar). The polycarbonate membranes (8-μm pore size) on the bottom of the upper compartment of the Transwells were coated with 1% human fibronectin (R&D Systems. catalog no. 1918-FN). The cells were harvested 24 hours after transfection and suspended in serum-free DMEM culture medium. Then, cells were added to the upper chamber (4 × 10^4^ cells/well). At the same time, 0.5 mL of DMEM containing 10% FBS was added to the lower compartment, and the Transwell-containing plates were incubated for 12 hours in a humidified atmosphere containing 5% CO_2_ atmosphere. After the incubation, cells that had invaded to the lower surface of the filter membrane were fixed with 4% paraformaldehyde for 25 min at room temperature, washed three times with distilled water, and stained with 0.1% crystal violet in 0.1 M borate and 2% ethanol for 15 min at room temperature. Cells remaining on the upper surface of the filter membrane (non-migratory) were gently scraped off using a cotton swab. Images of the lower surfaces (with the migratory cells) were captured by using a photomicroscope (5 fields/chamber) (BX51 Olympus, Japan), and the cells in randomly selected fields were counted by a blinded investigator.

### Construction of the miR-138-5p overexpression lentiviral vector

A lentiviral vector that overexpresses miR-138-5p was purchased from Invitrogen. When T24 cells reached 70% confluence in either 6-well plates or 100-mm dishes, lentivirus at a multiplicity of infection of 10 was added together with Polybrene (final concentration 5 μg/ml) according to the manufacturer’s instructions. Cells were then harvested for animal experiments.

### Establishment of Tumor Xenografts in Mice

Six-week-old male nude mice were purchased from the Model Animal Research Center at Nanjing University (Nanjing, China) and maintained under specific pathogen-free conditions at Nanjing University. The mice were randomly divided into 4 groups and subcutaneously injected with T24 cells (2 × 10^6^ cells per mouse, 5 mice per group, Fig. 4a) that were infected with either control lentivirus or a lentivirus that overexpressed miR-138-5p followed by co-transfection with a control plasmid or a BIRC5 overexpression plasmid. The needle was inserted into the left side of the armpit, midway down, at a depth of 5 mm deep an angle of 45°. The volume of the tumors was measured every 4 days after implantation. The tumor volume was calculated by the following formula: tumor volume [mm^3^] = (length [mm]) × (width [mm])^2^ × 0.52. The mice were sacrificed after 28 days. The tumors were excised, and the tumor weight was measured. Portions of the tumor samples were used for protein and total RNA extraction, and the remainder was fixed in 4% paraformaldehyde for 24 hours and then processed for hematoxylin and eosin (H&E) staining as well as immunohistochemical staining for Survivin and Ki-67.

### Statistical analysis

All of the Western blotting and cell invasion assay results are representative of at least three independent experiments. Quantitative RT-PCR and the luciferase reporter and cell proliferation assays were performed in triplicate, and each experiment was repeated several times. The data shown are the mean ± SD or mean ± SE of at least three independent experiments.

## Results

### The discordance of Survivin protein and mRNA level in bladder cancer tissues

**Fig. 1:**
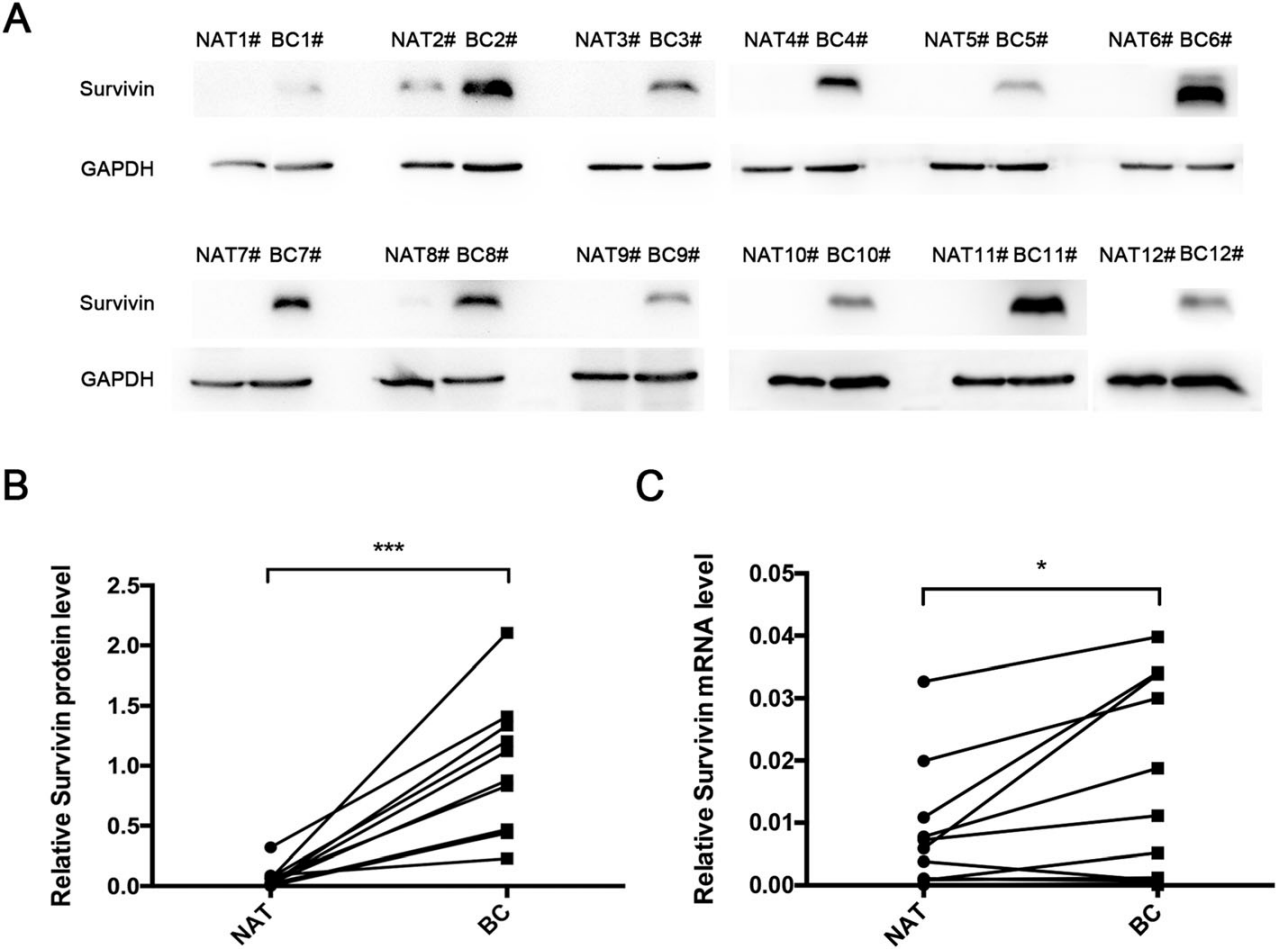
Survivin protein and mRNA expression levels in bladder cancer and paired normal adjacent tissues. (**a** and **b**) Western blotting analysis of the expression levels of Survivin protein in human bladder cancer specimens (BC) and normal adjacent tissues (NAT). **a**: representative Western blotting image; **b**: quantitative analysis. (**c**) Quantitative RT-PCR analysis of the relative expression of Survivin mRNA in human bladder cancer specimens and normal adjacent tissues. *p < 0.05; *** *p* < 0.005

We first determined the expression pattern of Survivin in bladder cancer tissues. After measuring the levels of Survivin protein in 12 pairs of bladder cancer tissues and corresponding noncancerous tissues via Western blotting, we found that Survivin was either absent or weakly expressed in normal adjacent tissues and significantly higher in the cancer tissues (Fig. 1a and b). Subsequently, we performed quantitative RT-PCR to measure the levels of BIRC5 mRNA in the same 12 pairs of cancerous and noncancerous tissues. We found that Survivin mRNA was detectable in normal adjacent tissues and that the mRNA levels did not differ as much (approximately 2 times) as the protein levels between the cancerous and noncancerous tissues (Fig. 1c). This disparity between the differences in the Survivin protein and mRNA levels in bladder tissues strongly suggests that a post-transcriptional mechanism may be involved in the regulation of Survivin.

### Identification of conserved miR-138-5p target sites within the 3′-UTR of BIRC5

**Fig. 2:**
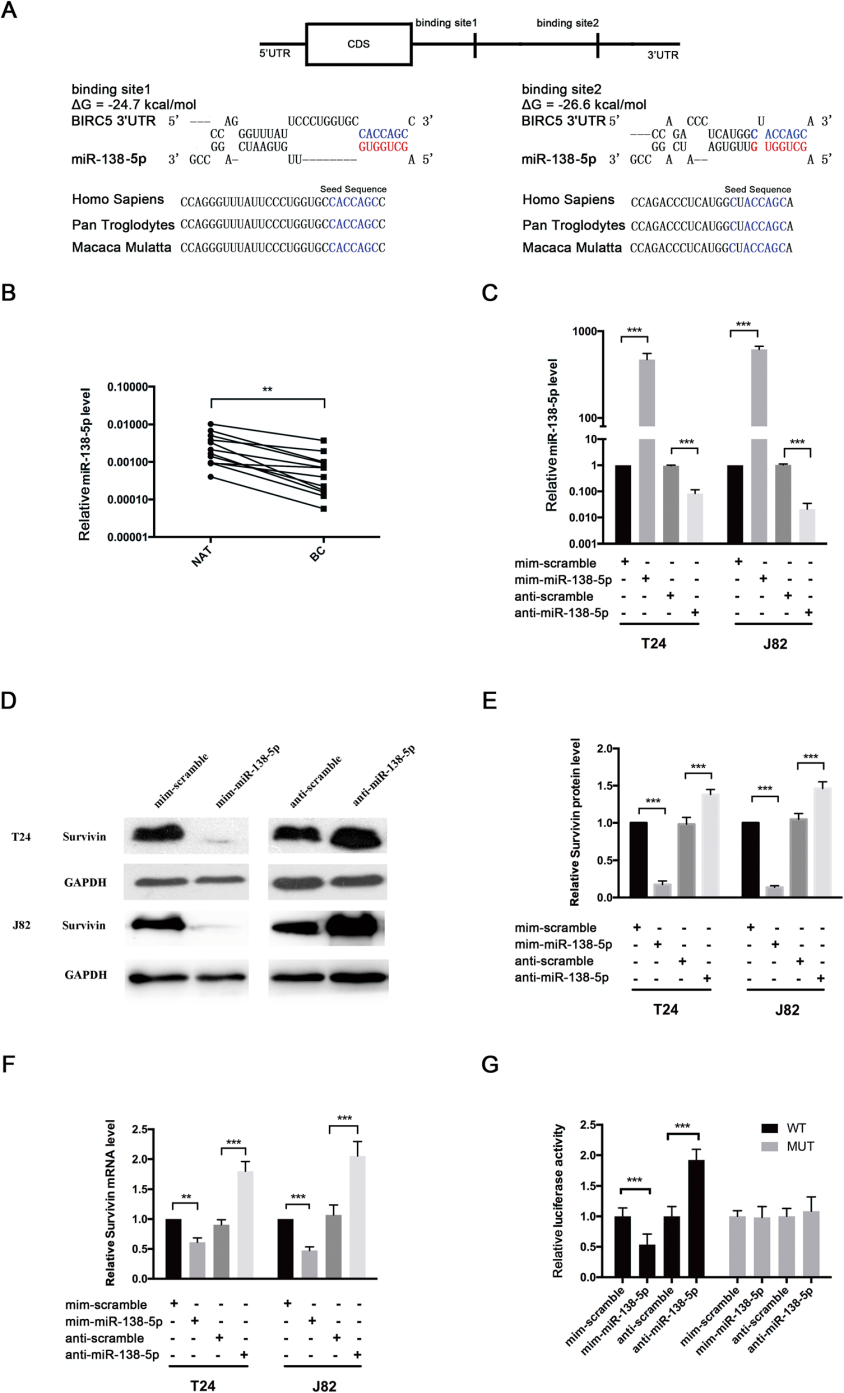
The negative regulation of Survivin expression by miR-138-5p in bladder cancer cells. **a** Schematic description of the hypothetical hybridizations formed by the interactions between the binding sites in the BIRC5 3′-UTR (*top*) and miR-138-5p (*bottom*). The seed regions of miR-138-5p and the seed-recognition sites in the BIRC5 3′-UTR are indicated in red. All the nucleotides in the seed-recognition sites are completely conserved in several species. The predicted free energy values of each hybrid are indicated. **b** Quantitative RT-PCR analysis of the relative expression levels (miR-138-5p vs. U6) of miR-138-5p in human bladder cancer specimens (BC) and normal adjacent tissues (NAT). **c** Quantitative RT-PCR analysis of the expression levels of miR-138-5p in T24 and J82 cells transfected with equal doses of the miR-138-5p mimic (mim-miR-138-5p), miR-138-5p inhibitor (anti-miR-138-5p) or corresponding scrambled negative control RNA (mim-scramble and anti-scramble, respectively). **d** and **e** Western blotting analysis to detect Survivin protein levels in T24 and J82 cells transfected with equal amounts of mim-miR-138-5p, anti-miR-138-5p, mim-scramble or anti-scramble. **d**: representative image; **e**: quantitative analysis. **f** Quantitative RT-PCR analysis of Survivin mRNA levels in T24 and J82 cells transfected with equal amounts of mim-miR-138-5p, anti-miR-138-5p, mim-scramble or anti-scramble. **g** Direct recognition of the Survivin 3’-UTR by miR-138-5p. Firefly luciferase reporters containing either wild-type (WT) or mutant (MUT) miR-138-5p binding sites in the Survivin 3′-UTR were co-transfected with equal doses of mim-miR-138-5p, anti-miR-138-5p or the corresponding scrambled negative control RNA (mim-scramble and anti-scramble, respectively) into HEK293 cells. At 24 hours after transfection, the cells were assayed using a luciferase assay kit. Firefly luciferase values were normalized to β-galactosidase activity, and the results were calculated as the ratio of firefly luciferase activity in the miR-138-5p-transfected cells normalized to the activity in the negative control RNA-transfected cells. The results are presented as the mean ± S.D. of three independent experiments. *p < 0.05; ** *p* < 0.01; *** *p* < 0.005

miRNAs post-transcriptionally regulate their target mRNA via sequence-guided recognition to trigger either mRNA degradation or translational repression. miRNAs are therefore likely to play a biologically relevant role in regulating Survivin expression in bladder cancer. Using three publicly available algorithms (TargetScan, miRanda and PicTar), miR-138-5p was identified as a candidate miRNA that could target BIRC5. The predicted interaction between miR-138-5p and its target sites in the BIRC5 3′-UTR is illustrated in Fig. 2a, which shows that miR-138-5p has 2 potential target sites in the 3′-UTR of the BIRC5 mRNA sequence. There is perfect base-pairing between the seed region (the core sequence that encompasses the first 2-8 bases of the mature miRNA) and the predicted target sites. The minimum free energy values of the two hybridization sites are -24.7 and -26.6 kcal/mol, respectively, which are well within the range of genuine miRNA-target pairs. Furthermore, the miR-138-5p binding sequences in the BIRC5 3′-UTR were highly conserved across multiple species.

### Detection of an inverse correlation between miR-138-5p and Survivin levels in bladder cancer tissues

Because the expression patterns of miRNAs are generally thought to be opposite to that of their targets, we next investigated whether miR-138-5p was inversely correlated with Survivin protein in bladder cancer. After determining the levels of miR-138-5p in the same 12 pairs of bladder cancer tissues and adjacent noncancerous tissues, we found that miR-138-5p levels were notably down-regulated in bladder cancer tissues (Fig. 2b). Thus, Survivin was deduced to be a miR-138-5p target not only by computational prediction but also based on the inverse correlation between miR-138-5p and Survivin protein levels in human bladder tissues.

### Validation of Survivin as a direct target of miR-138-5p

The correlation between miR-138-5p and Survivin was further examined by evaluating Survivin expression after either overexpressing or knocking down miR-138-5p in the human bladder cancer cell lines T24 and J82. Overexpression of miRNA was achieved by transfecting cells with mim-miR-138-5p, which is a synthetic RNA oligonucleotide that mimics the miR-138-5p precursor. Knockdown of miRNA was achieved by transfecting cells with anti-miR-138-5p, which is a chemically modified antisense oligonucleotide designed to specifically target mature miR-138-5p. The efficient overexpression and knockdown of miR-138-5p in T24 and J82 cells are shown in Fig. 2c. Cellular miR-138-5p levels were increased approximately 500-fold when T24 and J82 cells were transfected with mim-miR-138-5p, and these levels dropped to approximately 10% of the normal level when T24 and J82 were treated with anti-miR-138-5p. As predicted, overexpressing miR-138-5p significantly suppressed the Survivin protein levels in T24 and J82 cells, whereas miR-138-5p knockdown had the opposite effect on Survivin expression in these cells (Fig. 2d and e).

To determine how miR-138-5p influenced the expression of Survivin in bladder cancer, we repeated the abovementioned experiments and examined the expression of Survivin mRNA after transfection. The levels of BIRC5 mRNA were down-regulated upon miR-138-5p overexpression, whereas the levels of BIRC5 mRNA were up-regulated upon miR-138-5p knockdown (Fig. 2f). These results demonstrated that post-transcriptional regulation of Survivin mRNA by miR-138-5p may partially contribute to the mRNA degradation of this gene.

To determine whether the negative regulatory effects of miR-138-5p on Survivin expression were mediated through the binding of miR-138-5p to the presumed sites in the 3′-UTR of the Survivin mRNA, the full-length 3′-UTR of BIRC5 containing the 2 presumed miR-138-5p binding sites was cloned downstream of the firefly luciferase gene in a reporter plasmid. The resulting plasmid was transfected into HEK293 cells along with mim-miR-138-5p, anti-miR-138-5p, or corresponding scrambled negative control RNAs (mim-scramble and anti-scramble, respectively). As expected, luciferase activity was markedly reduced in the cells transfected with mim-miR-138-5p and increased in the cells transfected with anti-miR-138-5p (Fig. 2g). Furthermore, we introduced point mutations into the corresponding complementary sites in the 3′-UTR of Survivin to eliminate the predicted miR-138-5p binding sites (i.e., the two binding positions were mutated). This mutated luciferase reporter was unaffected by either overexpression or knockdown of miR-138-5p (Fig. 2g). This finding suggested that these binding sites strongly contribute to the interaction between miR-138-5p and Survivin mRNA. In conclusion, our results demonstrated that miR-138-5p directly recognizes and binds to the 3′-UTR of the Survivin mRNA transcript to inhibit Survivin translation in bladder cancer cells.

### The role of miR-138-5p in regulating Survivin in bladder cancer cells

Survivin is known to be involved in the regulation of cell proliferation and invasion in various cancer cells. In present study, we investigated the impact of Survivin on the proliferation and invasion of bladder cancer cells. To knock down Survivin, small interfering RNAs (siRNAs) targeting Survivin were designed and then transfected into T24 cells. To overexpress Survivin, an expression plasmid designed to specifically express the full-length ORF of Survivin without the miR-138-5p-responsive 3′-UTR was also constructed and transfected into T24 cells. Efficient knockdown and overexpression of Survivin in T24 cells are shown in Additional file 1: Figure S1 A–B. The CCK-8 assay was used to determine cell proliferation, while the transwell invasion assay was used to determine the invasive capability. T24 cells transfected with Survivin siRNA exhibited a reduction in cell proliferation and invasion; in contrast, transfection with the Survivin overexpression plasmid had the opposite effect on cell proliferation and invasion (Additional file 1: Figure S1 C–F).

**Fig. 3:**
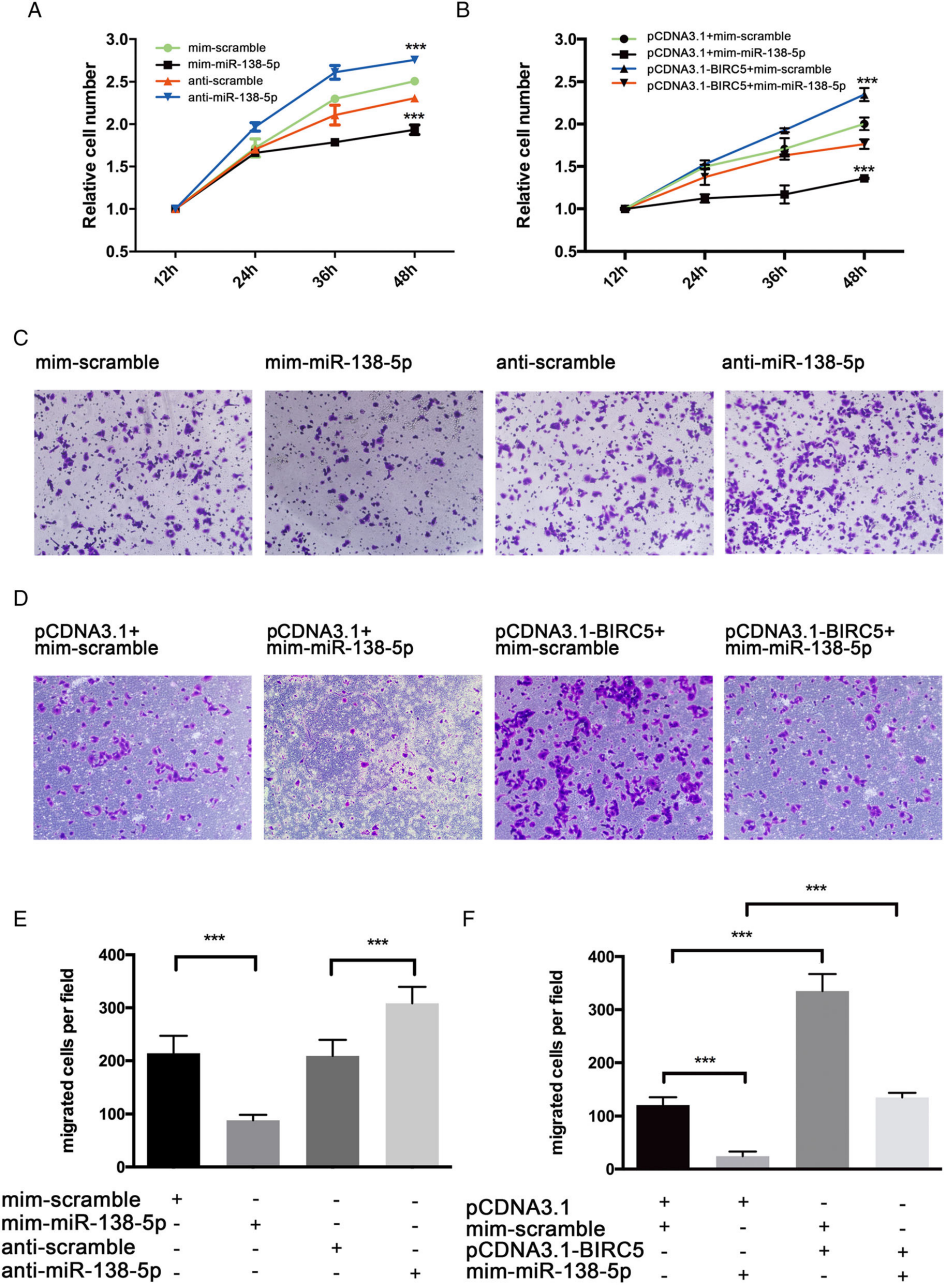
The role of miR-138-5p targeting Survivin in regulating the proliferative and invasive abilities of bladder cancer cells. **a** The CCK-8 viability assay was performed 12, 24, 36, and 48 hours after T24 cells were transfected with miR-138-5p mimic (mim-miR-138-5p), miR-138-5p inhibitor (anti-miR-138-5p) or corresponding scrambled negative control RNA (mim-scramble and anti-scramble, respectively). The viability of the experimentally treated cells were compared to that of their associated control-treated cells (i.e., mim-miR-138-5p vs mim-scramble; anti-miR-138-5p vs anti-scramble). **b** The CCK-8 viability assay was performed 12, 24, 36, and 48 hours after T24 cells were transfected with mim-scramble plus control vector (pCDNA3.1), mim-miR-138-5p plus control vector, mim-scramble plus BIRC5 plasmid (pCDNA3.1-BIRC5), or mim-miR-138-5p plus BIRC5 plasmid (pCDNA3.1 + mim-scramble vs pCDNA3.1-BIRC5 + mim-scramble, and pCDNA3.1 + mim-miR-138-5p vs pCDNA3.1-BIRC5 + mim-miR-138-5p, respectively). **c** and **e**, transwell analysis of invading T24 cells treated with equal amounts of mim-miR-138-5p, anti-miR-138-5p, mim-scramble or anti-scramble. (**c**): representative image; (**e**): quantitative analysis. **d** and (**f**), transwell analysis of invading T24 cells treated with equal amounts of mim-scramble plus control vector (pCDNA3.1), mim-miR-138-5p plus control vector, mim-scramble plus BIRC5 plasmid (pCDNA3.1-BIRC5), or mim-miR-138-5p plus BIRC5 plasmid. (**d**), representative image; (**f**), quantitative analysis. *p < 0.05; ** *p* < 0.01; *** *p* < 0.005

**Fig. 4:**
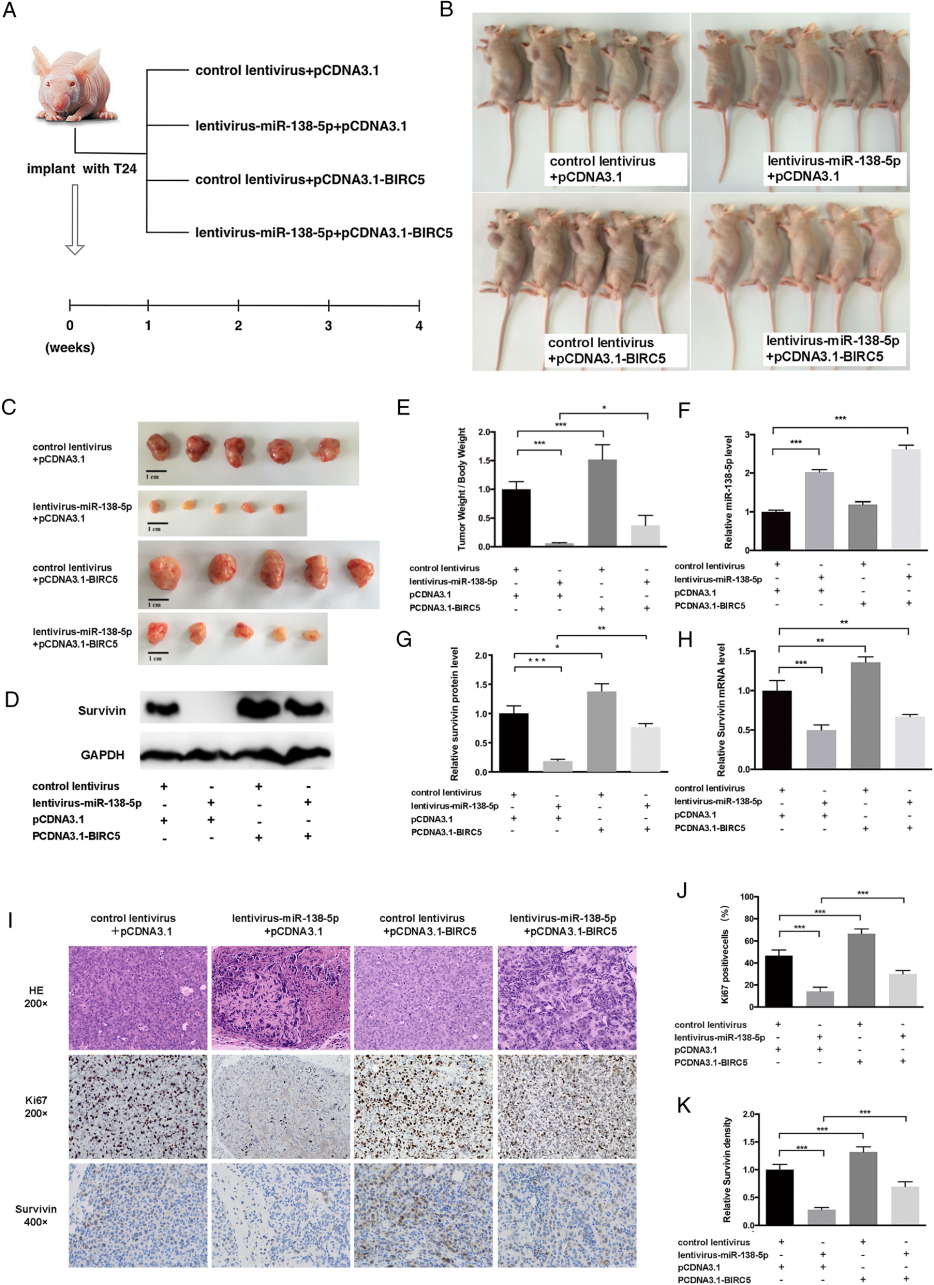
Effects of miR-138-5p and Survivin on the growth of bladder cancer cell xenografts in mice. (**a**) Overview of the experimental design. T24 cells were infected with either a control lentivirus or a lentivirus containing a plasmid that overexpressed miR-138-5p and then transfected with either a control plasmid or a BIRC5 overexpression plasmid. Each group of T24 cells (2 × 10^6^ cells per 0.1 mL) was subcutaneously injected into 6-week-old nude mice (5 mice per group), and tumor growth was evaluated on day 28 after cell implantation. (**b**) and (**c**), Representative images of the injected mice and excised tumors in the different groups. (**d**), Western blotting analysis of Survivin in tumors from injected mice. (**e**)-(**h**), Quantitative analysis of 4 parameters in tumors from the injected mice. (**e**), Quantitative analysis of the tumor weight, (**f**), Quantitative RT-PCR analysis of miR-138-5p. (**g**), Quantitative analysis of Survivin protein levels. (**h**), Quantitative analysis of Survivin mRNA levels. (I)-(K), H&E-stained sections and immunohistochemical staining for Ki-67 and Survivin in the tumors from injected mice. (**i**): representative images; (**j**)-(**k**): quantitative analysis (J. Ki-67-positive cells; K. Survivin density). *p < 0.05; ** *p* < 0.01; *** *p* < 0.005

We further analyzed the biological consequences of the miR-138-5p-driven repression of Survivin expression in bladder cancer cells. As expected, T24 cells transfected with mim-miR-138-5p showed decreased cell proliferation; in contrast, knocking down miR-138-5p had the opposite effect on cell proliferation (Fig. 3a). Moreover, compared with cells transfected with mim-miR-138-5p alone, T24 cells transfected with both mim-miR-138-5p and the Survivin overexpression plasmid exhibited significantly higher proliferation rates (Fig. 3b), suggesting that miR-138-5p-resistant Survivin is sufficient to rescue the suppression of Survivin by miR-138-5p and to attenuate the anti-proliferative effect of miR-138-5p on bladder cancer cells. We also evaluated the effects of miR-138-5p on the invasive ability of T24 cells using transwell invasion assays, which showed that the percentage of invasive cells was significantly lower among T24 cells transfected with mim-miR-138-5p and higher among cells transfected with anti-miR-138-5p (Fig. 3c and e). However, when T24 cells were co-transfected with mim-miR-138-5p and the Survivin overexpression plasmid, Survivin dramatically attenuated the suppressive effect of miR-138-5p on cell invasion (Fig. 4d and f). These results indicate that miR-138-5p might inhibit cell proliferation and invasion by silencing Survivin.

### The effect of miR-138-5p and Survivin on bladder tumor growth in vivo

We evaluated the effects of miR-138-5p and Survivin on the growth of bladder cancer cell xenografts in nude mice. A 300-bp fragment containing the genomic miR-138-5p sequence was cloned into a lentiviral expression plasmid and produced into lentivirus-miR-138-5p, which was used to infect T24 cells and overexpress miR-138-5p. T24 cells were infected with either a control lentivirus or lentivirus-miR-138-5p (MOI = 5); the resulting cells were then co-transfected with a control plasmid or a BIRC5 overexpression plasmid (6 μg/ 75 cm^2^) to produce 4 cellular phenotypes. These cells were subcutaneously injected into 4 different groups of 6-week-old nude mice (Fig. 4a). The growth curves of the tumors in the different groups are shown in the Additional file 2: Figure S2. After 28 days of growth *in vivo*, the mice were sacrificed, and the weight of the xenografted tumors was measured (Fig. 4, b and c). A significant decrease in the size and weight was observed in tumors from the miR-138-5p-overexpressing group compared to those of the control group, whereas the size and weight of the tumors in the group implanted with cells containing the BIRC5-overexpression plasmid were dramatically increased (Fig. 4, c and e). Additionally, BIRC5 overexpression attenuated the repressive effect of miR-138-5p on tumor growth (Fig. 4, c and e), suggesting that miR-138-5p might inhibit tumor growth by silencing Survivin. Subsequently, total RNA and proteins were isolated from the tumors and analyzed. After 28 days of xenograft growth *in vivo*, tumors from the miR-138-5p-overexpression group showed a significant increase in miR-138-5p expression compared to tumors from the control groups (Fig. 4f). Likewise, BIRC5 mRNA levels were increased in the tumors from the BIRC5-overexpressing group (Fig. 4h). Tumors from the miR-138-5p-overexpressing group displayed a reduction in the Survivin protein levels compared to tumors from the control group, whereas the tumors from the BIRC5-overexpressing group showed elevated Survivin protein levels. Tumors with both miR-138-5p and BIRC5 overexpression exhibited significantly higher levels of Survivin compared to tumors with miR-138-5p overexpression alone (Fig. 4, d and g), suggesting that BIRC5 overexpression could rescue the Survivin suppression caused by miR-138-5p. We also determined the level of BIRC5 mRNA in the tumors by using qPCR. The BIRC5 mRNA was significant reduced in the tumors with miR-138-5p overexpression, which was consistent with the *in vitro* results (Fig. 4h).

Furthermore, hematoxylin and eosin (H&E) staining of xenograft tissues showed confluent necrotic areas and reduced cell mitosis in the group implanted with the cells expressing the miR-138-5p lentiviral vector compared with the control group, whereas an increase in cell mitosis was observed in the xenografts from the BIRC5 overexpression group (Fig. 4i). Xenografts with both miR-138-5p and BIRC5 overexpression exhibited increased cell mitosis compared to xenografts with only miR-138-5p overexpression (Fig. 4i), suggesting that Survivin overexpression could attenuate the anti-proliferative effect of miR-138-5p. Immunohistochemical staining also revealed the presence of lower levels of Survivin in tumors from mice implanted with miR-138-5p-overexpressing cells, whereas the tumors from the BIRC5-overexpressing mice showed increased Survivin protein levels. Tumors with both miR-138-5p and BIRC5 overexpression exhibited increased Survivin protein levels compared to xenografts with only miR-138-5p overexpression (Fig. 4i and k). Finally, the proliferative activity of the tumor cells was assessed by immunocytochemistry with the mouse monoclonal antibody targeting Ki-67. The cell proliferation rate as indicated by the percentage of Ki-67-positive tumor cells was increased in the group implanted with cells containing the BIRC5 plasmid and decreased in the group implanted with cells containing the miR-138-5p lentiviral vector. Likewise, BIRC5 overexpression attenuated the pro-proliferative effect caused by miR-138-5p overexpression (Fig. 4, i and j). These results were consistent with the findings of the *in vitro* assays, which firmly validated the role of miR-138-5p as a tumor suppressor by targeting BIRC5.

## Discussion

Survivin is an oncogene that regulates the apoptosis, proliferation, and invasion of many cancers, including bladder cancer [16–19]. Survivin has been recognized as a highly specific biomarker for bladder cancer and its expression is relative to the presence, stage, progression and mortality of bladder cancer [20]. As a tumor biomarker, Survivin protein is highly expressed in bladder tumors and either absent or weakly expressed in the normal adjacent bladder mucosa [21]. Interestingly, we found that the Survivin mRNA was detectable in normal bladder tissue and did not differ as much as the protein levels between bladder cancer and normal adjacent bladder mucosa. The discordance between Survivin protein and mRNA in bladder cancer suggested that post-transcriptional regulation might be involved in Survivin protein expression. One essential mode of post-transcriptional regulation is the repression of mRNA transcripts by miRNA. miRNAs regulate gene expression by the sequence-selective targeting of mRNAs, leading to either translational repression or mRNA degradation [8, 22].

It was reported that miRNAs related to post-transcriptional regulation play an important role in Survivin dysregulation in some human cancers [13]. However, there is limited information about the miRNA regulation of Survivin expression in bladder cancer. In this study, we searched for miRNAs that can target Survivin and identified miR-138-5p as a candidate. We experimentally validated the direct inhibition of Survivin translation by miR-138-5p by overexpressing and knocking down miR-138-5p in bladder cancer cells. In addition, we showed that in cultured bladder cancer cells, miR-138-5p inhibited Survivin expression as well as cell proliferation and invasion; furthermore, miR-138-5p also slowed tumor growth in a xenograft mouse model. The results demonstrated a novel regulatory network involving miR-138-5p and Survivin to fine-tune the proliferation and invasion of bladder cancer.

miRNAs are aberrantly expressed during the carcinogenesis of bladder cancer [23, 24]. Some microRNAs have been categorized as “oncomiRs” as opposed to “tumor suppressor miRs” [25–27]. In this study, we found that the levels of miR-138-5p in bladder cancer were much lower than those in normal adjacent bladder mucosa. Down-regulation of miR-138-5p has also been reported in other cancers [28–30]. All these results suggested that miR-138-5p may work as a tumor suppressor in bladder cancer. It is well known that a single miRNA can target multiple genes, whereas multiple miRNAs can target a single gene. For example, miR-138-5p could inhibit the translation of ZEB2 mRNA and suppress the ZEB2-mediated metastatic potential of bladder cancer [31]. miR-138-5p could also suppress cell proliferation by targeting Bag-1 in gallbladder carcinoma [32]. To investigate the role of this new pathway in the network of bladder carcinogenesis signaling, we overexpressed miR-138-5p in bladder cancer cells and found that proliferation and invasion of bladder cancer cells were inhibited, which mimic the function of Survivin reduction by targeted siRNA. Interestingly, we further observed that the restoration of Survivin expression by an overexpressing plasmid can successfully attenuate the anti-proliferative and anti-invasive effect of miR-138-5p on bladder cancer cells, although miR-138-5p may also have many other targets. In the *in vivo* study, the Survivin overexpression plasmid significantly rescued the suppressed tumor growth induced by cells transduced with lentivirus-miR-138-5p. As is common knowledge, the effect of the plasmid did not last as long as that of the lentiviral vector, which was one limitation of our study. Therefore, the tumors in the rescue group did not grow to the level of the control group as expected. In the following supplemental animal experiment, we increased the dose of Survivin overexpression plasmid, and the tumors in rescue group almost reached the levels of tumors in control group (~80%) (Additional file 3: Figure S3). These results confirmed that targeting Survivin is an important mechanism of the tumor-suppressive function of miR-138-5p.

Many studies have shown that Survivin could serve as a molecular target for bladder cancer therapy [33–35]. Knockdown of Survivin by using siRNA could dramatically suppress the proliferation and invasion of bladder cancer cells. As previously mentioned, post-transcriptional regulation by miRNA plays an important part in dysregulating Survivin signaling in bladder cancer [13]. Modulating the miRNA activities may provide exciting opportunities for bladder cancer therapy. The rationale for using miRNAs as therapeutic agents is based on the two following criteria: (1) miRNA expression is dysregulated in cancer compared to that in normal tissues and (2) the cancer phenotype can be adjusted by targeting miRNA expression. Emerging evidence suggests that either inhibiting the overexpression of oncogenic miRNAs or introducing more tumor-suppressive miRNAs could become a novel treatment strategy in cancer therapy [36]. Compared to other strategies, miRNA-based therapeutics have several advantages; for example, miRNAs as therapeutic agents have the ability to target multiple genes, frequently in the context of a network [37]. In the present study, we found that elevated expression of Survivin was related to the decrease of tumor suppressor miR-138-5p. Treatment with miR-138-5p exhibited an anti-tumor effect both *in vitro* and *in vivo* by negatively regulating Survivin expression. Thus, it is hypothesized that a replacement treatment with miR-138-5p mimics could be a promising strategy for cancers characterized by down-regulation of miR-138-5p.

## Conclusions

In summary, this study revealed a critical role of miR-138-5p as a tumor suppressive miRNA in bladder carcinogenesis, explored the molecular mechanisms by which aberrant miR-138-5p expression contributes to bladder cancer progression and identified Survivin as a direct target of miR-138-5p. Regulation of Survivin by miR-138-5p might explain why the decrease of miR-138-5p during bladder carcinogenesis can promote cancer progression. This study may provide a new strategy for future bladder cancer therapies.

## Additional files

Additional file 1: Figure S1. The role of siRNAs targeting Survivin and a Survivin overexpression plasmid in regulating the proliferative and invasive activities of bladder cancer cells. (A) Western blotting analysis to detect Survivin protein levels in T24 cells transfected with siRNAs targeting Survivin. (B) Western blotting analysis to detect Survivin protein levels in T24 cells transfected with the Survivin overexpression plasmid. (C) The CCK-8 viability assay was performed 12, 24, 36, and 48 hours after the transfection of T24 cells with si-Survivin#1. (D) The CCK-8 viability assay was performed 12, 24, 36, and 48 hours after the transfection of T24 cells with the Survivin overexpression plasmid. (E) and (F) Transwell analysis of invading T24 cells treated with si-Survivin#1. E: representative image; F: quantitative analysis. (G) and (H) Transwell analysis of invading T24 cells treated with the Survivin overexpression plasmid. G: representative image; H: quantitative analysis. *** p < 0.005. (TIF 5210 kb)
Additional file 2: Figure S2. The growth curves of the bladder cancer cell xenografts. (TIFF 207 kb)
Additional file 3: Figure S3. Effects of miR-138-5p and Survivin on the growth of bladder cancer cell xenografts in mice (supplemental animal experiment). A. Western blotting analysis of Survivin protein levels in T24 cells transduced with either lentivirus-miR-138-5p (MOI = 5) and/or different doses of the Survivin overexpression plasmid. B, Representative images of the tumors in different groups. The MOI value of lentivirus-miR-138-5p used here was 5, and the amount of pCDNA3.1-BIRC5 transfected was 9 μg/75 cm^2^. C, Quantitative analysis of the tumor weights. **p* < 0.05; ** *p* < 0.01; *** *p* < 0.005. (TIF 5960 kb)

## Abbreviations

3′-UTR: 3′ untranslated region
BC: Bladder cancer
CCK-8: Cell Counting Kit-8
FBS: Fetal bovine serum
H&E: Hematoxylin and eosin
IAP: Inhibitor of apoptosis protein
miRNA: microRNA
MOI: Multiplicity of infection
NAT: Normal adjacent tissues
ORF: Open reading frame
RT-PCR: Reverse transcription polymerase chain reaction
siRNA: small interfering RNA
β-gal: β-galactosidase
