# Supplementary figures and images for: miR-138-5p contributes to cell proliferation and invasion by targeting Survivin in bladder cancer cells
Source: Mol Cancer. 2016 Dec 15;15:82. doi: 10.1186/s12943-016-0569-4 (PMC5159976; doi:10.1186/s12943-016-0569-4)

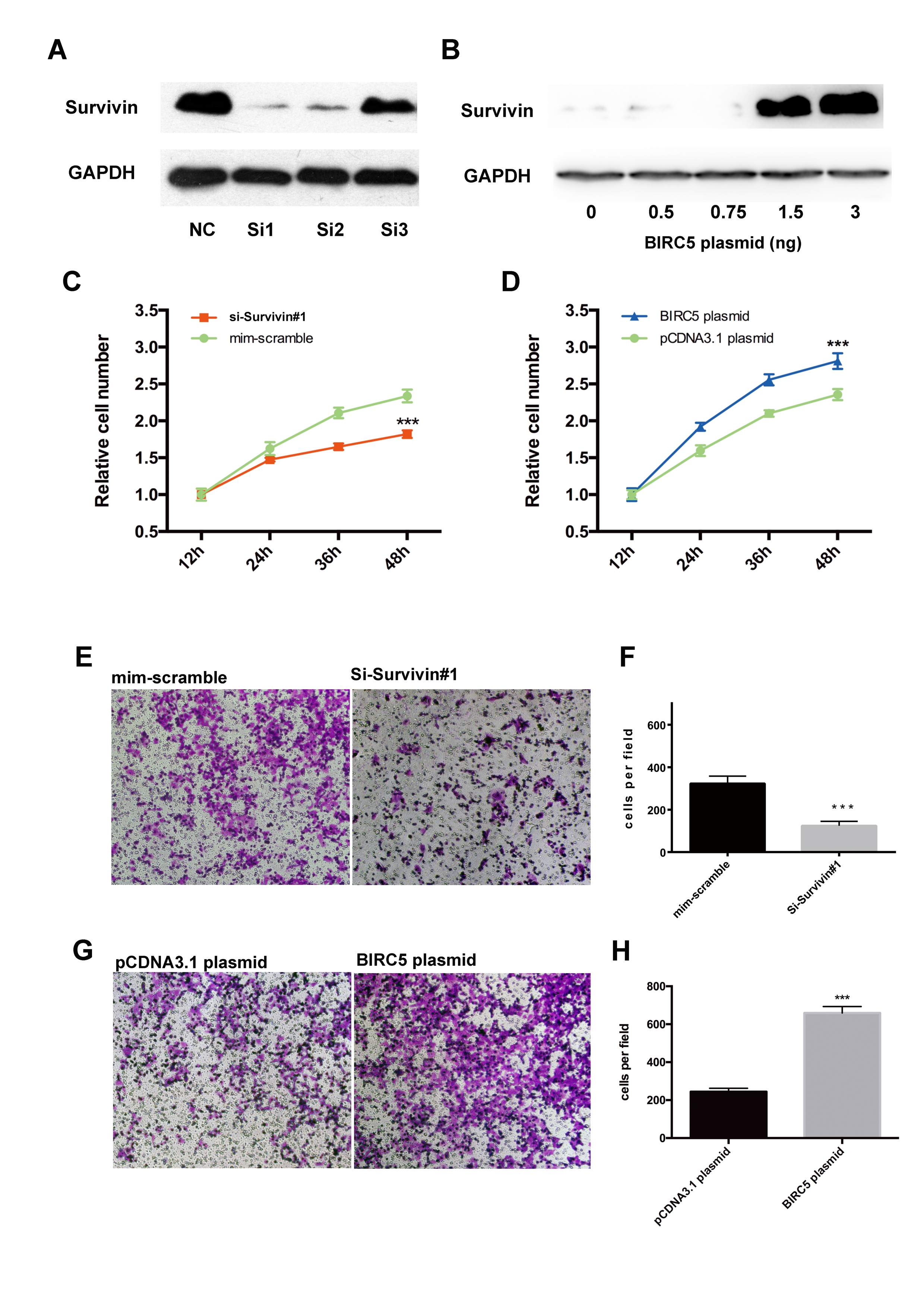

Supplement: Additional file 1: Figure S1. — The role of siRNAs targeting Survivin and a Survivin overexpression plasmid in regulating the proliferative and invasive activities of bladder cancer cells. (A) Western blotting analysis to detect Survivin protein levels in T24 cells transfected with siRNAs targeting Survivin. (B) Western blotting analysis to detect Survivin protein levels in T24 cells transfected with the Survivin overexpression plasmid. (C) The CCK-8 viability assay was performed 12, 24, 36, and 48 hours after the transfection of T24 cells with si-Survivin#1. (D) The CCK-8 viability assay was performed 12, 24, 36, and 48 hours after the transfection of T24 cells with the Survivin overexpression plasmid. (E) and (F) Transwell analysis of invading T24 cells treated with si-Survivin#1. E: representative image; F: quantitative analysis. (G) and (H) Transwell analysis of invading T24 cells treated with the Survivin overexpression plasmid. G: representative image; H: quantitative analysis. *** p < 0.005. (TIF 5210 kb) [file 12943_2016_569_MOESM1_ESM.tif]

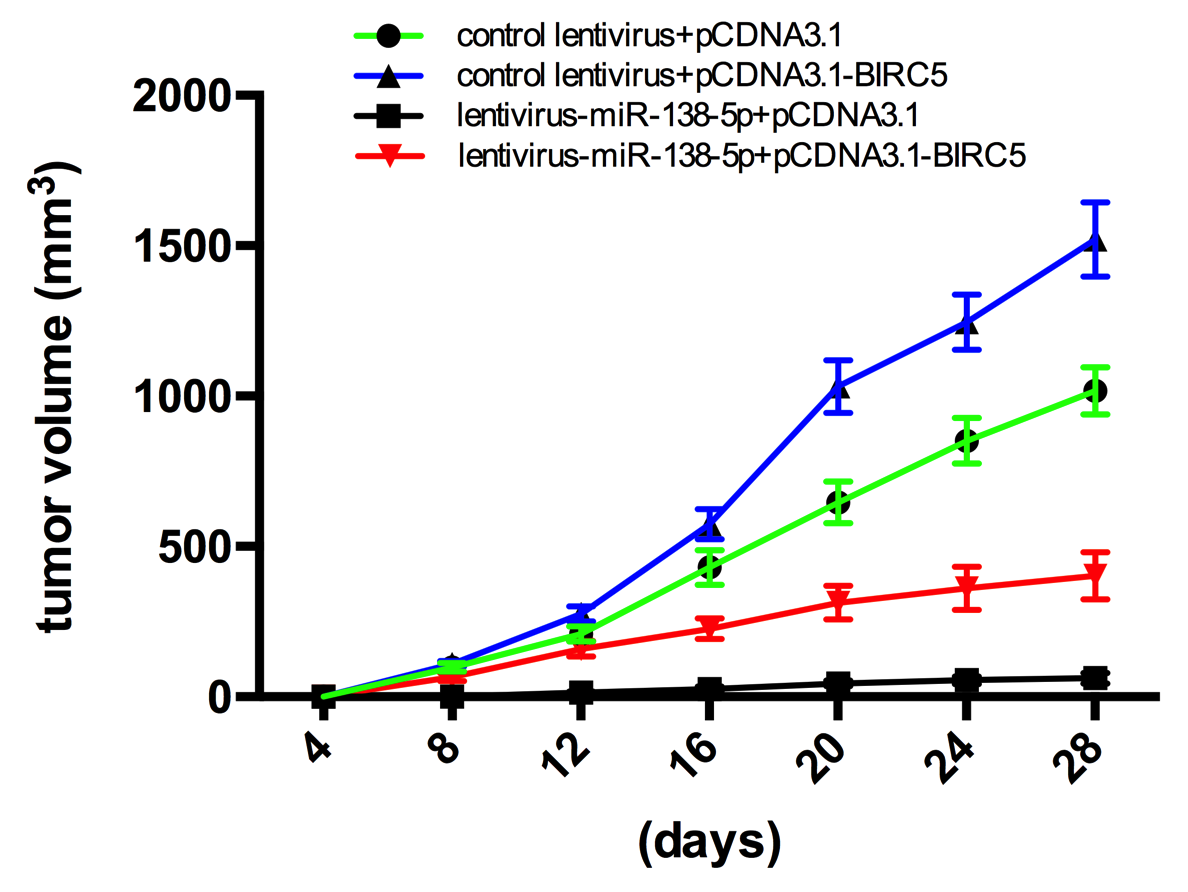

Supplement: Additional file 2: Figure S2. — The growth curves of the bladder cancer cell xenografts. (TIFF 207 kb) [file 12943_2016_569_MOESM2_ESM.tiff]

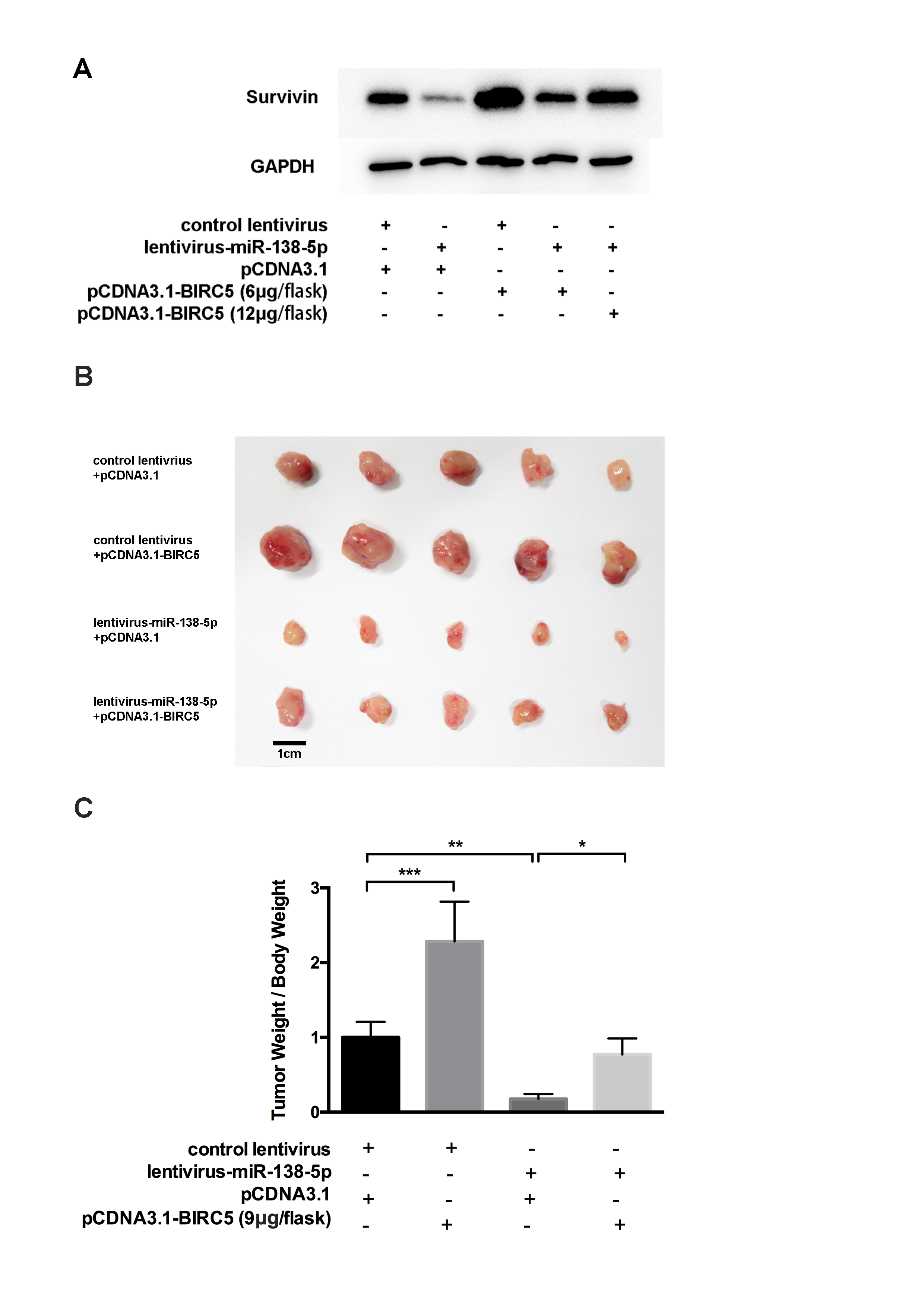

Supplement: Additional file 3: Figure S3. — Effects of miR-138-5p and Survivin on the growth of bladder cancer cell xenografts in mice (supplemental animal experiment). A. Western blotting analysis of Survivin protein levels in T24 cells transduced with either lentivirus-miR-138-5p (MOI = 5) and/or different doses of the Survivin overexpression plasmid. B, Representative images of the tumors in different groups. The MOI value of lentivirus-miR-138-5p used here was 5, and the amount of pCDNA3.1-BIRC5 transfected was 9 μg/75 cm2. C, Quantitative analysis of the tumor weights. *p < 0.05; ** p < 0.01; *** p < 0.005. (TIF 5960 kb) [file 12943_2016_569_MOESM3_ESM.tif]
